# FDG–PET in the prediction of survival of patients with cancer of the pancreas: a pilot study

**DOI:** 10.1054/bjoc.2000.1166

**Published:** 2000-07-03

**Authors:** N R Maisey, A Webb, G D Flux, A Padhani, D C Cunningham, R J Ott, A Norman

**Affiliations:** Department of Medicine, Nuclear Medicine and Academic Department of Diagnostic Radiology, The Royal Marsden NHS Trust, Sutton, Surrey, SM2 5PT, UK

## Abstract

Carcinoma of the pancreas is an aggressive tumour with an extremely poor prognosis. Recent studies have shown that chemotherapy can improve survival as well as quality of life. Since the prognosis is generally poor, the identification of early responders to chemotherapy is important to avoid unnecessary toxicity in patients who are not responding. Response assessment by conventional radiographic methods is problematical because treatment induces fibrosis and makes tumour measurements difficult. The aim of this pilot study was to assess 18-fluoro-deoxy-glucose positron emission tomography (FDG-PET) as an early marker of the benefit of chemotherapy. Eleven patients with histologically proven adenocarcinoma of the pancreas were treated with protracted venous infusional 5-fluorouracil (PVI 5-FU) alone or PVI 5-FU and mitomycin C (MMC). FDG-PET scans were performed prior to and at 1 month following the commencement of chemotherapy. FDG uptake was compared with the tumour dimensions measured on a computer tomographic (CT) scan. Patients were followed up for relapse, death and symptomatic response. Three of the 11 patients had no measurable FDG uptake prior to chemotherapy. Of the eight patients who had measurable uptake prior to treatment, seven had a reduction in uptake at 1 month. Six out of the 11 patients had no measurable FDG uptake at 1 month. The overall survival (OS) in these patients ranged from 124 to 1460 days, with a median of 318.5 days. This was superior in comparison to patients who had residual FDG uptake at 1 month (median survival 318.5 days vs 139 days;*P* = 0.034) and there was a trend to improved symptoms (84% [5/6] vs 20% [1/5];*P* = 0.13). There was no statistically significant correlation between best CT response and FDG uptake at 1 month. These results suggest that the absence of FDG uptake at 1 month following chemotherapy for carcinoma of the pancreas is an indicator of improved overall survival. This suggests that FDG-PET may be superior to response assessment by conventional radiographic methods and FDG-PET may have the potential to help make difficult treatment decisions in the management of pancreatic cancer. Larger prospective studies are required to confirm this finding. © 2000 Cancer Research Campaign


British Journal of Cancer (2000) **83**(3), 287–293
© 2000 Cancer Research Campaign
doi: 10.1054/ bjoc.2000.1166, available online at http://www.idealibrary.com on IDEAL®

### FDG–PET in the prediction of survival of patients with cancer of the pancreas: a pilot study

**NR Maisey, A Webb, GD Flux, A Padhani, DC Cunningham, RJ Ott and A Norman**

Department of Medicine, Nuclear Medicine and Academic Department of Diagnostic Radiology, The Royal Marsden NHS Trust, Sutton, Surrey SM2 5PT, UK

**Summary** Carcinoma of the pancreas is an aggressive tumour with an extremely poor prognosis. Recent studies have shown that chemotherapy can improve survival as well as quality of life. Since the prognosis is generally poor, the identification of early responders to chemotherapy is important to avoid unnecessary toxicity in patients who are not responding. Response assessment by conventional radiographic methods is problematical because treatment induces fibrosis and makes tumour measurements difficult. The aim of this pilot study was to assess 18-fluoro-deoxy-glucose positron emission tomography (FDG-PET) as an early marker of the benefit of chemotherapy. Eleven patients with histologically proven adenocarcinoma of the pancreas were treated with protracted venous infusional 5-fluorouracil (PVI 5-FU) alone or PVI 5-FU and mitomycin C (MMC). FDG-PET scans were performed prior to and at 1 month following the commencement of chemotherapy. FDG uptake was compared with the tumour dimensions measured on a computer tomographic (CT) scan. Patients were followed up for relapse, death and symptomatic response. Three of the 11 patients had no measurable FDG uptake prior to chemotherapy. Of the eight patients who had measurable uptake prior to treatment, seven had a reduction in uptake at 1 month. Six out of the 11 patients had no measurable FDG uptake at 1 month. The overall survival (OS) in these patients ranged from 124 to 1460 days, with a median of 318.5 days. This was superior in comparison to patients who had residual FDG uptake at 1 month (median survival 318.5 days vs 139 days; $P = 0.034$) and there was a trend to improved symptoms (84% [5/6] vs 20% [1/5]; $P = 0.13$). There was no statistically significant correlation between best CT response and FDG uptake at 1 month. These results suggest that the absence of FDG uptake at 1 month following chemotherapy for carcinoma of the pancreas is an indicator of improved overall survival. This suggests that FDG-PET may be superior to response assessment by conventional radiographic methods and FDG-PET may have the potential to help make difficult treatment decisions in the management of pancreatic cancer. Larger prospective studies are required to confirm this finding. © 2000 Cancer Research Campaign

Carcinoma of the pancreas is the cause of death in approximately 7000 patients each year in the UK (Black et al, 1997). At diagnosis, fewer than 20% of patients have lesions suitable for potentially curative surgical resection. However, even in this favourable group, the 5-year survival is only around 10% (Ahlgren et al, 1992). Of the patients who are inoperable, the median survival time is 4–6 months (Prott et al, 1997). Palliative chemotherapy is generally offered to fit patients (performance status of 2 or less). This approach is justified by two recent studies comparing chemotherapy and best supportive care (BSC). Palmer et al (1994) found a median overall survival of 8 months in patients treated with FAM chemotherapy (5-FU (5-fluorouracil), adriamycin and mitomycin-C) compared with 3.5 months in patients receiving BSC. Glimelius et al (1996) randomized 93 patients with inoperable pancreatic and biliary carcinoma to receive chemotherapy or BSC and found a median overall survival of 6 months in the chemotherapy group, with a median overall survival of 2.5 months in the BSC arm. They also reported an improved quality of life (QoL) in the treatment arm. Other groups have investigated the impact of chemotherapy on QoL. At the Royal Marsden Hospital, Nicolson et al (1995) found an objective radiological response in 16% of patients treated with infusional 5-FU and cisplatin with 34% of patients reporting an improved performance status (PS). In particular, 60% had a reduction in pain, 70% reported a reduction in nausea and vomiting and 91% of patients had a reduction in symptoms of reflux. Burris et al (1997) found 24% of patients receiving gemcitabine had a clinical benefit (defined as a decrease in pain and analgesic intake, increase in weight and improved performance status), compared with 4.8% in patients treated with 5-FU ($P = 0.0022$). The objective response, however, was only 5.4% in the gemcitabine arm as compared with 0% for the 5-FU arm. The median survival was 5.65 months with gemcitabine and 4.41 months with 5-FU ($P = 0.0025$). Thus it can be seen that objective response, as assessed by conventional radiographic techniques, may not always correlate with patient survival or symptomatic response. Objective evaluation of response in pancreatic cancer is often difficult. Pancreatic tumours less than 3 cm often cause minimal distortion of the pancreatic architecture and may remain iso-dense to normal pancreatic tissue. A proportion of tumours are diffuse and have irregular, indistinct tumour borders. There is often associated necrosis, abcess and pseudocyst formation, and biopsies often reveal a mixture of tumour cells, inflammatory tissue and fibrosis (Wittenburg et al, 1982). What is required is an early predictor of response in order to avoid any unnecessary toxicity, and to assist in a rational treatment plan.

Positron emission tomography (PET) using 18-fluoro-deoxy-glucose (FDG) is becoming an increasingly important tool in the management of various cancers. Tumours show enhanced utilization of glucose compared with normal tissue as a result of a

*Received 2 November 1998*
*Revised 22 November 1999*
*Accepted 17 February 2000*

*Correspondence to:* DC Cunningham number of factors, including up-regulation of hexokinase, decreased glucose-6-phosphatase activity and changes in the levels of Glut membrane transporters, especially Glut 1 (Higashi et al, 1997; Reske et al, 1997). Higashi et al, found raised Glut-1 expression in 26 out of 28 malignant pancreatic tumours (88%). The uptake of FDG is similar to that of glucose but, due to differences in the further metabolic profile (including slow dephosphorylation compared to glucose), there is enhanced intracellular retention. FDG-PET has been used in the detection and staging of various cancers and has proven benefit for the detection of recurrent disease. A number of small studies have looked at the effect of therapy induced changes in FDG uptake. Finlay et al (1996) at this centre investigated the response to chemotherapy of liver metastases in patients with colorectal cancer. They found that the 4- to 5-week tumour to normal liver (T:L) ratio was able to discriminate response from non-response both in a lesion-by-lesion and overall patient response assessment. Other studies have substantiated these results (Wahl et al, 1991; Okazumi et al, 1992; Haberkorn et al, 1993; Hoekstra et al, 1993; Okada et al, 1994*a*). A recent European PET oncology workshop examined the collective experience of several European centres. It concluded that changes in the uptake of FDG following one or two cycles of chemotherapy, compared with pretreatment uptake, may be able to predict clinical outcome and that the greater the decrease in post-therapy uptake, the better the response (Price and Jones, 1995). FDG-PET has also been used in the evaluation of pancreatic cancer. Bares et al (1994) reported that they were able to detect 24 out of 27 pancreatic cancers on visual assessment of FDG-PET scans in patients who had pancreatic masses on CT scan, where they took focal FDG uptake to represent malignancy. They found a positive predictive value (PPV) for detecting cancer of 92%, and a negative predictive value (NPV) of 85%. Quantification (by calculation of the differential-uptake-ratio or ‘DAR’) did not improve their interpretation over visual assessment. Zimny et al (1997) assessed 106 cases of pancreatic mass with FDG-PET. Visual interpretation gave them 63 out of 74 true positives, and 27 out of 32 true negatives. The overall sensitivity was 85%, with a specificity of 84%. In euglycaemic patients they found the sensitivity of PET to be 98% in detecting pancreatic cancer. Inokuma et al (1995) found that FDG-PET in histologically proven pancreatic cancer gave a sensitivity of 96%, both qualitatively by visual assessment (focal FDG uptake representing malignancy) and quantitatively (by calculation of the standardized-uptake-value or ‘SUV’).

Objective response in pancreatic cancer by conventional radiographic methods is often problematical; the aim of this pilot study was to investigate the feasibility of FDG-PET in assessing the clinical benefit of chemotherapy. Due to the well-documented poor correlation between conventional radiological techniques and response, overall survival is the optimum measure of the benefit of treatment. Therefore the correlation between FDG uptake following the commencement of chemotherapy and survival was taken as the primary end point of this study. In addition the correlation between symptomatic response and radiological response was examined as secondary end points.

### MATERIALS AND METHODS

### Patients

Patients were recruited prospectively over an 8-month period between March and November 1995. Five patients were treated with infusional 5-FU alone and six patients were treated with infusional 5-FU and MMC. This was part of a study protocol approved by our Institutional committees on clinical research and patient ethics. Every 6 weeks, symptoms were recorded using a protocol trial questionnaire. FDG-PET scans were performed prior to, and at 1 month following, the start of therapy. CT scans were performed prior to treatment, and at 1, 3, 6, 12 and 24 months following the start of treatment and patients were assessed for response according to WHO criteria (Miller et al, 1981). Personnel reading the CT scans were blinded to the clinical and PET data. After completion of treatment patients were followed up 3-monthly in the outpatient department and monitored for signs of relapse. Relapse was defined using standard clinical and radiological criteria. The elapsed time from finishing treatment to relapse and/or death was recorded.

### FGD-PET

List mode data were acquired on the MUP-PET positron camera. This consists of two large area multiwire proportional chambers mounted on a rotating gantry (Marsden et al, 1989). These data were backprojected into a $64^3$ matrix, with cubic voxel dimensions of length 0.6 cm. Back-projected images were attenuation and scatter corrected and deconvolved with an experimentally determined point spread function. The MUP-PET positron camera has an axial-field-of view (FoV) of 30 cm but in order to satisfy the spatial invariance criterion needed for the three-dimensional backprojection and deconvolution image reconstruction method only the central 15 cm of this axial FoV was used here. A reconstructed spatial resolution of 6 mm can be achieved when imaging a point source but, due to the low camera sensitivity, a Hanning filter with a frequency cut-off of 0.4 $cm^{-1}$ was used in this study. With this filter the spatial resolution (FWHM) is ~2 cm. This clearly would have an effect on the absolute quantification of small tumours due to the partial volume effect.

Patients were fasted for at least 4 h prior to the scan. An administration of 50–150 MBq $^{18}$FDG was given 1 h prior to the scan. Scans were acquired for 30–45 min, enabling 1.5 million coincidence events to be collected. Patients were reproducibly positioned on the couch using a laser initially focused on the xiphisternum.

Where uptake corresponded to radiographic abnormality, region of interest (ROI) analysis was carried out on a volume of $4 \times 4 \times 1$ voxels (0.216 $cm^3$) surrounding the area of maximum intensity on each scan. The whole lesion was not defined due to the indeterminacy of delineating the boundary. Defining a region of maximum intensity enabled volumes to be compared consistently between studies. Background values were determined from undefined tissue obtained contra-laterally where ROIs were again of the same volume as for the tumour ROIs.

Tumours were located on the PET images by reference to the known anatomical location at the centre of the field of view. Tumours were also localized by their spatial relationship to the kidneys. To aid tumour localization, image registration with CT data was carried out for five patients using a point-based method with in-house software (Flux, 1995). For this, external markers were used, comprising of perspex discs containing a cavity which was filled with Ge-68 for the FDG scans and with a solution of $BaCl_2$ for the CT. In those cases where image registration was carried out, the average accuracy was 8 mm between the PET and CT, determined as the rms (root mean square) distance between corresponding pairs of markers.

© 2000 Cancer Research Campaign

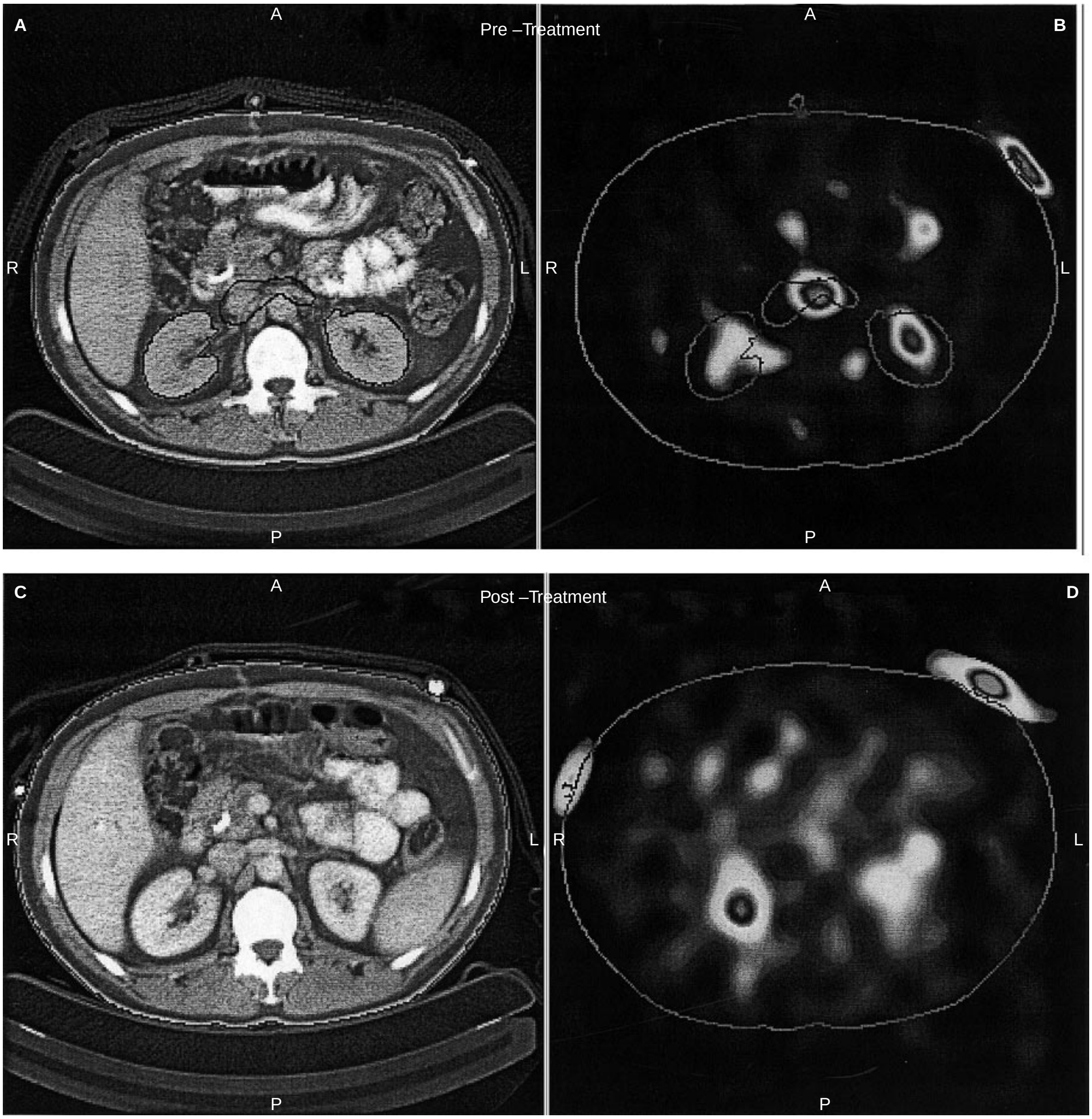

**Figure 1** A 34-year-old female with adenocarcinoma of the pancreas. (**A**) Pretreatment CT demonstrating a large mass in the head of the pancreas. (**B**) Pretreatment FDG-PET scan showing physiological FDG renal uptake and FDG uptake in the area of the pancreatic mass seen on the CT. (**C**) CT scan after 1 month of 5-FU and MMC chemotherapy with partial response by CT criteria. (**D**) FDG-PET scan after 1 month of chemotherapy shows continued metabolic activity in the tumour. The ROI analysis shows no significant decrease in FDG uptake in the tumour despite an apparent visual decrease – see Table 2

Personnel reading the PET scans were blinded to the clinical and radiological response and survival data.

This study was approved by our Institutional committees on clinical research and patient ethics, and received ARSAC certification.

### Statistical analysis

Categorical data were examined using $\chi^2$ test. Fisher's Exact test was used where expected cell counts were less than five. Survival curves were calculated using the methods of Kaplan and Meier, and compared using the log-rank test.

### RESULTS

Eleven patients (seven men and four women) with histologically proven adenocarcinoma of the pancreas participated in this study. The ages ranged from 34 to 69 years, with a median of 57 years, and a mean of 54.4 years. Patient data are listed in Table 1. During the period of the study all patients relapsed and all but one patient died. The overall survival (OS) ranged from 84 days to 1460 days (1 patient alive at the time writing) with a median of 242 days. The progression-free survival (PFS) ranged from 49 to 497 days, with a median of 123 days.

### PET response (Table 2)

Of the 11 patients who had FDG-PET scans, eight had a tumour-to background (T/B) ratio prior to treatment greater than 1. One patient had increased uptake in the pancreas but the volume was too small to extract reliable statistics. Eight patients demonstrated a reduction in FDG uptake a month after the start of treatment, and six patients had no measurable FDG uptake at this time. One

*© 2000 Cancer Research Campaign*

**Table 1** Patient data

| | |
|---|---|
| No. of patients | 11 |
| Median age, years | 57 |
| Sex | |
| Male | 7 |
| Female | 4 |
| Treatment | |
| PVI 5-FU | 5 |
| PVI 5-FU and MMC | 6 |
| Locally advanced | 6 |
| Metastatic | 5 |
| Size of primary (mm) | |
| Range | 20–130 |
| Mean | 52.4 |

patient had an increase in FDG uptake at 1 month (Figure 1). The apparent visual decrease in uptake is due to the tumour intensity relative to the kidneys in that slice. Objective ROI analysis however does show an increase in tumour/background activity as given in Table 3. However, within the errors of the measurements, this is compatible with no fall in glucose uptake.

The T/B values quoted in Table 2 may be considered to be semi-quantitative in that they quantify the relative uptake between pre- and post-therapy scans of a patient but do not give absolute uptake values.

The median OS in the 8 patients who had a PET response was 225 days (range 84–1460 days). The median PFS was 99.5 days (range 49–468 days). The median OS in the patients with no reduction in FDG uptake was 245 days (range 139–553 days), and the median PFS was 175 days (range 123–497 days). However, two of the three patients who had no FDG uptake reduction, had no FDG uptake prior to treatment suggesting low metabolic activity.

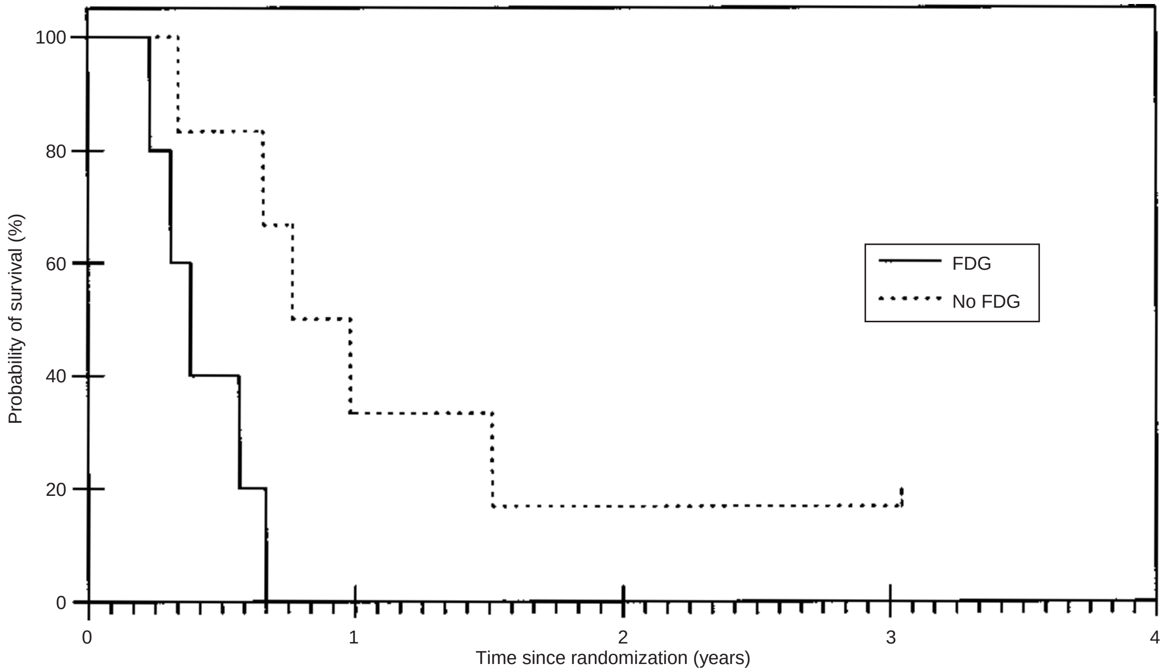

**Figure 2** The survival curves of patients with or without FDG uptake at 1 month after the start of treatment. This figure demonstrates that patients who had no uptake at 1 month had a statistically significant increased overall survival compared to patients with residual uptake (*P* = 0.034)

**Table 2** Pre- and post-treatment tumour-to-background values ± s.d.

| Patient no. | Pretreatment tumour-to-background ratio | Post-treatment tumour-to-background ratio | Ratios Pre/Post |
|---|---|---|---|
| 1 | 1.40±0.12 | 1.83±0.32 | 0.76±0.15 |
| 2 | 3.25±1.00 | 2.66±0.66 | 1.22±0.48 |
| 3 | 1.77±0.45 | 1.45±0.45 | 1.22±0.48 |
| 4 | 2.18±0.25 | 1.72±0.35 | 1.27±0.30 |
| 5 | 1.81±0.26 | 1.15±0.20 | 1.58±0.36 |
| 6 | 1.77±0.54 | 1.00 | NA |
| 7 | 1.87±0.24 | 1.00 | NA |
| 8 | 1.88±0.32 | 1.00 | NA |
| 9 | [a] | 1.00 | NA |
| 10 | 1.0 | 1.00 | NA |
| 11 | 1.0 | 1.00 | NA |

[a] Positive, but volume too small to extract reliable statistics.

 © 2000 Cancer Research Campaign

**Table 3** Patient treatment, CT response and survival

| Patient no. | Age | Sex | Metastatic disease | Site | Size (mm) | Chemotherapy | CT response at 1/12 | Best CT response | PFS (days) | OS (days) |
|---|---|---|---|---|---|---|---|---|---|---|
| 1 | 34 | F | No | Head | 45×60 | 5FU+MMC | PR | PR | 123 | 139 |
| 2 | 53 | M | Yes | Tail | 130×110 | 5FU+MMC | SD | SD | 106[a] | 106 |
| 3 | 69 | M | Yes | Head | 30×40 | 5FU+MMC | SD | SD | 49 | 84 |
| 4 | 47 | F | No | Head | 42×55 | 5FU+MMC | SD | SD | 86 | 208 |
| 5 | 59 | M | No | Head | 50×30 | 5FU+MMC | SD | PR | 202 | 242 |
| 6 | 38 | M | Yes | Head | 30×45 | 5FU | SD | PR | 468 | [b] |
| 7 | 63 | F | Yes | Head | 20×31 | 5FU | SD | SD | 78 | 124 |
| 8 | 52 | M | No | Head | 40×40 | 5FU+MMC | SD | PR | 257 | 280 |
| 9 | 57 | F | No | Head | 30×35 | 5FU | SD | SD | 93 | 357 |
| 10 | 62 | M | Yes | Head | 50×40 | 5FU | SD | SD | 175 | 245 |
| 11 | 65 | M | No | Head | 28×40 | 5FU | SD | SD | 497 | 553 |

[a] Patient no. 2 died from a gastrointestinal bleed. [b] Patient still alive at time of writing.

**Table 4** FDG, CT and symptomatic response

| | FDG uptake at 1/12 | No FDG uptake at 1/12 |
|---|---|---|
| Symptomatic response | 1 | 4 |
| No Symptomatic response | 5 | 1 |
| | *P* = 0.134 (Two-tailed Fisher's exact test) | |
| CT response | 2 | 3 |
| No CT response | 2 | 4 |
| | *P* = 0.81 (one-tailed Fisher's exact test) | |

In the six patients who had no detectable activity at 1 month, the median OS was 318.5 days (range 124–1460 days). The median PFS was 216 days (range 78–497 days). In the patients who had residual FDG uptake at 1 month the median OS was 139 days (range 84–242 days). The median PFS was 106 days (range 49–202 days). There was a statistically significant difference in median OS between those patients who had no FDG uptake at one month compared with those patients who had residual activity ($P = 0.034$) (Figure 2)

### Radiological response (Table 3)

Of the 11 patients, only one patient had a radiological response at the time of the post-treatment PET scan at 1 month (compared with seven who had a PET response). Three further patients had a radiological response by CT criteria, all at 3 months from the commencement of treatment. In all, four patients had a radiological partial response (PR) to treatment. Of these four patients, three also had a reduction in FDG uptake following treatment. Of the three patients who had either an increase or no change in the FDG uptake following therapy, only one had a radiological partial response. In patients with a radiological response the median PFS was 335 days (range 123–468 days). The median OS was 261 days (range 139–1460 days). In patients with no radiological response the median PFS was 78 days (range 49–497 days). The OS was 208 days (range 84–553 days). There was no statistically significant correlation between best CT response and FDG uptake at 1 month. Radiological response did not significantly predict for OS or PFS at 1 month.

### Symptomatic response (Table 4)

Five of the six patients who had no FDG uptake at 1 month post-treatment reported a subjective improvement in their symptoms following treatment. Four of these patients had abdominal pain that resolved completely following therapy. Three out of these six patients became completely asymptomatic on therapy, having previously reported symptoms ranging from reflux oesophagitis to abdominal pain. The one patient who had no change in their symptoms initially reported only reflux oesophagitis, which continued despite treatment.

Only one of the five patients (patient 5) who had continuing FDG uptake at 1 month reported an improvement of their symptoms. Interestingly this patient had a large drop in FDG uptake following treatment with a pre-/post-treatment ratio of 1.6. The remaining four patients reported no improvement in their symptoms. There appears to be a trend towards correlation between FDG uptake at 1 month and symptoms that fails to reach statistical significance ($P = 0.134$).

### DISCUSSION

Objective monitoring of the effectiveness of new cancer treatments is essential if these treatments are to become widely acceptable. The ability to predict therapeutic response early during a course of treatment for pancreatic cancer is important since prognosis is poor, lifespan limited and toxicity in non-responding patients is not acceptable. Currently, CT is the accepted method of assessing therapeutic response of pancreatic cancer but has a number of important limitations. These include the inability to clearly outline tumour borders for measurement purposes in responding patients and complex anatomical relationships. Furthermore CT is anatomical and yields no information on tumour metabolism or on the functional effects of treatment (e.g. vascular shut-down, apoptosis etc). FDG-PET has previously been shown to be a useful imaging technique in assessing response to treatment in other cancers.

© 2000 Cancer Research Campaign 

Our results show that following treatment there was a reduction in FDG uptake in seven out of the 11 patients studied. In this patient group there was a median survival of 242 days and an overall response rate (using radiological criteria) of 36% (four out of 11 responders) which when compared to historical data of patients receiving best supportive care only, suggests a favourable response to treatment. Interestingly, of the seven patients who had a reduction in FDG uptake in their post-therapy PET scan, only three had a CT response overall and only one at the time of the PET scan. This implies that FDG PET may be superior as an early predictor of response than CT. In addition there was no statistically significant correlation between best CT response and FDG uptake at 1 month following the commencement of treatment.

At 1 month following the start of treatment six patients had no detectable FDG uptake. This patient group had a statistically significant improved OS ($P = 0.019$) when compared to patients with residual FDG uptake. The improved median survival in-patients with no FDG uptake at 1 month is not explained by the absence of metastatic disease. Four of the six patients with no FDG uptake had metastatic disease, compared with none of the patients with residual FDG uptake.

Five of the six patients with no FDG uptake had a symptomatic improvement, compared with only one of the five patients with residual activity. This is not explained by different toxicity profiles of the two chemotherapeutic regimens, since previous work has shown no significant difference in toxicity between 5-FU alone and 5-FU with MMC (Ross et al, 1997). Although this result did not reach statistical significance ($P = 0.134$) the trend is encouraging.

One other interesting possibility to arise from this study is that pretreatment PET scans may be able to predict for tumour behaviour. Three patients had no measurable FDG uptake in their pretreatment scan, and had an OS of 357, 245 and 553 days respectively (the median survival of all patients being 242 days). It would be a reasonable assumption that low uptake of FDG correlates with low metabolic activity, and as such may represent a favourable prognostic factor.

Previous work has demonstrated that pretreatment FDG PET scans in different tumour types may be able to predict tumour behaviour and prognosis. However, many of these studies suffer from small numbers and a heterogeneity of histological grades as well as treatment options. Okada et al (1994*b*) reported a statistically significant decreased OS in patients with a high DAR in their series of 34 patients with lymphoma. However, treatment varied between radiotherapy (RT), chemotherapy, or a combination of both. They also studied a variety of different histological subgroups. Ahuja et al (1998) performed a retrospective study of 155 patients with newly diagnosed non-small-cell lung carcinoma prior to treatment. They reported that a low SUR (standardized uptake ratio) was predictive for prolonged survival. Again treatments varied, with patients with stage I–IIIa being eligible for complete resection, whereas stage IIIb–IV received chemotherapy or RT. In their series of 70 patients with primary breast cancer, Oshida et al (1998) reported that DAR was an independent predictor of relapse-free survival. However, 58 patients received a mastectomy and 12 had breast conserving therapy. Other groups have reported less convincing data. Minn et al (1997) found in their series of 37 patients with squamous cell head and neck cancer, the only independent prognostic factors were mitotic count and stage, despite there being a significantly improved 3-year OS in patients with a low SUV. Nakata et al (1997) studied a series of 14 patients with adenocarcinoma of the pancreas. They found a statistically significant prolonged survival in patients with a low SUV. However, treatments were extremely varied with only one patient receiving chemotherapy. A recent study by Higashi et al (1999) examined the effect of intraoperative radiation therapy (IORT) on FDG uptake in patients with adenocarcinoma of the pancreas. Their results indicated that the measurement of the average SUV in the tumour area could evaluate the local response of pancreatic cancer after IORT earlier and more markedly than with CT. However, they did not find a correlation with prognosis in these patients. One explanation may be that only eight of the 12 patients in this series received systemic treatment with chemotherapy. Changes in FDG uptake may therefore have reflected local control of the disease rather than overall survival. In the present study, although the numbers are small, all the patients have identical histology and all patients received chemotherapy.

Whilst recognizing the potential of PET it is important not to forget its limitations. Inflammatory tissue is recognized as a cause of false-positive results. In particular, chronic pancreatitis, cystadenoma, retroperitoneal fibrosis and lymphocyte infiltration may lead to false-positive results in patients with pancreatic masses (Bares et al, 1994; Inokuma et al, 1995). Despite this problem, studies have demonstrated a high PPV of FDG-PET (Bares et al, 1994; Zimny et al, 1997) suggesting that despite the occurrence of FP results, FDG-PET still has the ability to successfully identify malignant lesions. Treatment-induced changes (including RT and chemotherapy) have also been reported as a cause of false-positive results (Strauss, 1996). There are also a number of possible causes of false-negative results. FDG uptake has been shown to correlate with tumour grade, and low grade histology may be a cause of false-negative results (Rigo et al, 1996). Finally the sensitivity of the scanner will obviously have an influence on the rate of false-negative results.

There are a number of limitations of this study. As a pilot study the number of patients in this study is low and so although the results are statistically significant, the statistical power is low. The timing of the scans following therapy was chosen arbitrarily and may not be optimal. It would be useful to perform a series of scans to address this issue. Previous studies (Bares et al, 1994; Zimny et al, 1997) have reported the sensitivity of FDG-PET in the detection of pancreatic cancer to be 85–93%. The present study gave a sensitivity of 73%. Although the difference in sensitivity between these studies may in part be due to small numbers, the sensitivity of the MUP-PET cannot equal that of a modern commercial scanner. Due to the low camera sensitivity the uptake of FDG in small regions of tumour may be underestimated and this may effect the measurement of the level of response to treatment if the volume of the tumour changes substantially. However, in this study, only relative quantification was used to assess tumour response and, as most tumour sizes were greater than the spatial resolution (Table 3), partial volume effects are unlikely to substantially change the results.

Blood glucose was not routinely measured prior to PET scanning. However, although raised blood glucose has been reported as a cause of FN results, Zimney et al (1997) found no significant difference in the SUV between euglycaemic and hyperglycaemic patients in the 74 patients studied with histologically proven adenocarcinoma of the pancreas. Stollfuss et al (1995) found that of the 4 patients with pancreatic cancer who had FN findings, none had diabetes or raised blood glucose.

Although attempts were made to optimize reproducibility and positioning of the patients between studies FDG-PET scans, it

© 2000 Cancer Research Campaign must be remembered that this is a difficult area in PET and it is a possible source of error in any PET study.

In this study we have demonstrated a reduction in FDG uptake in seven out of 11 patients with pancreatic cancer following the commencement of chemotherapy, and that in the majority of patients this predates any response (if any) as assessed by CT scanning. Six patients had no FDG uptake at 1 month post treatment and these patients had a statistically significant advantage in OS compared to those with residual FDG uptake and possible correlation to symptomatic improvement. The results of this study are compatible with the limited published data and suggest that FDG-PET may a useful tool in assessing early response to treatment and predicting prognosis in pancreatic cancer.

### REFERENCES

Ahlgren JD, Hill MA and Roberts I (1992) *Pancreatic Cancer: Patterns, Diagnosis, and Approach to Treatment. Gastrointestinal Oncology*. JB Lippincott, Philadelphia

Ahuja V, Coleman RE, Herndon J and Patz EF Jr (1998) The prognostic significance of fluorodeoxyglucose positron emission tomography imaging for patients with non-small cell lung carcinoma. *Cancer* **83**: 918–924

Bares R, Klever P, Hauptmann S, Hellwig D, Fass J, Cremerius U, Schumpelick V, Mittermayer C and Bull U (1994) F-18 fluorodeoxyglucose PET in vivo evaluation of pancreatic glucose metabolism for detection of pancreatic cancer. *Radiology* **192**: 79–86

Black RJ, Bray F, Ferlay J and Parkin DM (1997) Cancer incidence and mortality in the European Union: cancer registry data and estimates of national incidence for 1990. *Eur J Cancer* **33**: 1057–1107

Burris HA, 3rd, Moore MJ, Andersen J, Green MR, Rothenberg ML, Modiano MR, Cripps MC, Portenoy RK, Storniolo AM, Tarassoff P, Nelson R, Dorr FA, Stephens CD and Von Hoff DD (1997) Improvements in survival and clinical benefit with gemcitabine as first-line therapy for patients with advanced pancreas cancer: a randomized trial. *J Clin Oncol* **15**: 2403–2413

Findlay M, Young H, Cunningham D, Iveson A, Cronin B, Hickish T, Pratt B, Husband J, Flower M and Ott R (1996) Non-invasive monitoring of tumor metabolism using fluorodeoxyglucose and positron emission tomography in colorectal cancer liver metastases: correlation with tumor response to fluorouracil [see comments]. *J Clin Oncol* **14**: 700–708

Flux GD (1995) *Multimodality image registration and its application to the dosimetry of intralesional radionuclide therapy*. University of London, London

Glimelius B, Hoffman K, Sjoden PO, Jacobsson G, Sellstrom H, Enander LK, Linne T and Svensson C (1996) Chemotherapy improves survival and quality of life in advanced pancreatic and biliary cancer. *Ann Oncol* **7**: 593–600

Haberkorn U, Strauss LG, Dimitrakopoulou A, Seiffert E, Oberdorfer F, Ziegler S, Reisser C, Doll J, Helus F and van Kaick G (1993) Fluorodeoxyglucose imaging of advanced head and neck cancer after chemotherapy. *J Nucl Med* **34**: 12–17

Higashi T, Tamaki N, Honda T, Torizuka T, Kimura T, Inokuma T, Ohshio G, Hosotani R, Imamura M and Konishi J (1997) Expression of glucose transporters in human pancreatic tumors compared with increased FDG accumulation in PET study. *J Nucl Med* **38**: 1337–1344

Higashi T, Sakahara H, Torizuka T, Nakamoto Y, Kanamori S, Hiraoka M, Imamura M, Nishimura Y, Tamaki N, Konishi J (1999) Evaluation of intraoperative radiation therapy for unrespectable pancreatic cancer with FDG PET. *J Nucl Med* **40**: 1424–1433

Hoekstra OS, Ossenkoppele GJ, Golding R, van Lingen A, Visser GW, Teule GJ and Huijgens PC (1993) Early treatment response in malignant lymphoma, as determined by planar fluorine-18-fluorodeoxyglucose scintigraphy. *J Nucl Med* **34**: 1706–1710

Inokuma T, Tamaki N, Torizuka T, Fujita T, Magata Y, Yonekura Y, Ohshio G, Imamura M, Konishi J, Bares R, Klever P, Hauptmann S, Hellwig D, Fass J, Cremerius U, Schumpelick V, Mittermayer C and Bull U (1995) Value of fluorine-18-fluorodeoxyglucose and thallium-201 in the detection of pancreatic cancer. *J Nucl Med* **36**: 229–235

Marsden PK, Ott RJ, Bateman JE, Cherry SR, Flower MA and Webb S (1989) The performance of a multiwire proportional chamber positron camera for clinical use. *Phys Med Biol* **34**: 1043–1062

Miller AB, Hoogstraten B, Staquet M and Winkler A (1981) Reporting results of cancer treatment. *Cancer* **47**: 207–214

Minn H, Lapela M, Klemi PJ, Grenman R, Leskinen S, Lindholm P, Bergman J, Eronen E, Haaparanta M and Joensuu H (1997) Prediction of survival with fluorine-18-fluoro-deoxyglucose and PET in head and neck cancer. *J Nucl Med* **38**: 1907–1911

Nakata B, Chung YS, Nishimura S, Nishihara T, Sakurai Y, Sawada T, Okamura T, Kawabe J, Ochi H and Sowa M (1997) 18F-fluorodeoxyglucose positron emission tomography and the prognosis of patients with pancreatic adenocarcinoma. *Cancer* **79**: 695–699

Nicolson M, Webb A, Cunningham D, Norman A, M, OB, Hill A and Hickish T (1995) Cisplatin and protracted venous infusion 5-fluorouracil (CF)-good symptom relief with low toxicity in advanced pancreatic carcinoma. *Ann Oncol* **6**: 801–804

Okada J, Oonishi H, Yoshikawa K, Imaseki K, Uno K, Itami J and Arimizu N (1994*a*) FDG-PET for the evaluation of tumor viability after anticancer therapy. *Ann Nucl Med* **8**: 109–113

Okada J, Oonishi H, Yoshikawa K, Itami J, Uno K, Imaseki K and Arimizu N (1994*b*) FDG-PET for predicting the prognosis of malignant lymphoma. *Ann Nucl Med* **8**: 187–191

Okazumi S, Isono K, Enomoto K, Kikuchi T, Ozaki M, Yamamoto H, Hayashi H, Asano T and Ryu M (1992) Evaluation of liver tumors using fluorine-18-fluorodeoxyglucose PET: characterization of tumor and assessment of effect of treatment [see comments]. *J Nucl Med* **33**: 333–339

Oshida M, Uno K, Suzuki M, Nagashima T, Hashimoto H, Yagata H, Shishikura T, Imazeki K and Nakajima N (1998) Predicting the prognoses of breast carcinoma patients with positron emission tomography using 2-deoxy-2-fluoro[18F]-D-glucose. *Cancer* **82**: 2227–2234

Palmer KR, Kerr M, Knowles G, Cull A, Carter DC and Leonard RC (1994) Chemotherapy prolongs survival in inoperable pancreatic carcinoma. *Br J Surg* **81**: 882–885

Price P and Jones T (1995) Can positron emission tomography (PET) be used to detect subclinical response to cancer therapy? The EC PET Oncology Concerted Action and the EORTC PET Study Group. *European Journal of Cancer* **31A**: 1924–1927

Prott FJ, Schonekaes K, Preusser P, Ostkamp K, Wagner W, Micke O, Potter R, Sulkowski U, Rube C, Berns T and Willich N (1997) Combined modality treatment with accelerated radiotherapy and chemotherapy in patients with locally advanced inoperable carcinoma of the pancreas: results of a feasibility study. *Br J Cancer* **75**: 597–601

Reske SN, Grillenberger KG, Glatting G, Port M, Hildebrandt M, Gansauge F and Beger HG (1997) Overexpression of glucose transporter 1 and increased FDG uptake in pancreatic carcinoma. *J Nucl Med* **38**: 1344–1348

Rigo P, Paulus P, Kaschten BJ, Hustinx R, Bury T, Jerusalem G, Benoit T and Foidart Willems J (1996) Oncological applications of positron emission tomography with fluorine-18 fluorodeoxyglucose. *Eur J Nucl Med* **23**: 1641–1674

Ross P, Norman A, Cunningham D, Webb A, Iveson T, Padhani A, Prendiville J, Watson M, Massey A, Popescu R and Oates J (1997) A prospective randomised trial of protracted venous infusion 5-fluorouracil with or without mitomycin C in advanced colorectal cancer. *Ann Oncol* **8**: 995–1001

Stollfuss JC, Glatting G, Friess H, Kocher F, Berger HG and Reske SN (1995) 2-(fluorine-18)-fluoro-2-deoxy-D-glucose PET in detection of pancreatic cancer: value of quantitative image interpretation [see comments]. *Radiology* **195**: 339–344

Strauss LG (1996) Fluorine-18 deoxyglucose and false-positive results: a major problem in the diagnostics of oncological patients. *Eur J Nucl Med* **23**: 1409–1415

Wahl R, Cody R, Zasadny K and al e (1991) Active breast cancer chemohormonotherapy sequentially assessed by FDG-PET: Early metabolic decrements precede tumour shrinkage. *J Nucl Med* **32**: 982

Wittenburg J, Simeone J and Ferrucci J (1982) Non-focal enlargement in pancreatic carcinoma. *Radiology* **144**: 131–135

Zimny M, Bares R, Fass J, Adam G, Cremerius U, Dohmen B, Klever P, Sabri O, Schumpelick V and Buell U (1997) Fluorine-18 fluorodeoxyglucose positron emission tomography in the differential diagnosis of pancreatic carcinoma: a report of 106 cases. *Eur J Nucl Med* **24**: 678–682

© 2000 Cancer Research Campaign
